# Ipsilateral Quadriceps Tendon-Bone ACL Revision Reconstruction to Address Failed Primary Bone-Patellar Tendon-Bone ACL Reconstruction with Persistent Patellar Bone Defect

**DOI:** 10.1016/j.eats.2022.11.032

**Published:** 2023-03-23

**Authors:** Iftach Hetsroni, Yuval Fuchs, Gideon Mann, Nissim Ohana

**Affiliations:** Department of Orthopedic Surgery, Meir General Hospital, Kfar Saba, affiliated to Sackler Faculty of Medicine, Tel Aviv University, Tel Aviv, Israel

## Abstract

Bone–patellar tendon–bone (BPTB) autograft is a popular graft choice for anterior cruciate ligament reconstruction (ACLR) in active young adults. In case of BPTB ACLR failure, the 3 most popular autograft choices for a revision surgery include contralateral BPTB, contralateral or ipsilateral hamstrings autograft, and contralateral or ipsilateral quadriceps tendon autograft. Quadriceps tendon autograft has been gaining increasing popularity in recent years in this respect, but using quadriceps tendon–bone autograft in the setup of a previous use of ipsilateral BPTB autograft deserves special technical considerations, with emphasis on preserving patellar bone integrity. We describe a technique for performing revision ACLR after failed primary BPTB ACLR by using ipsilateral quadriceps tendon–bone autograft in the setup of persistent distal patellar bone defect. Using this autograft benefits the advantages of highly resilient graft tissue in addition to fast bone-to-bone healing on the femoral side, and it can be an excellent choice in revision reconstruction for surgeons who prefer tendon-bone autograft for highly active young adults and specifically when the patients underwent bilateral primary autologous BPTB ACLRs.

Bone–patellar tendon–bone (BPTB) autograft is a popular graft choice for primary anterior cruciate ligament reconstruction (ACLR) in active adults due to low revision rates.^1^ In case of ruptured BPTB graft, the dilemma of graft choice is emphasized in revision surgery. While allograft can be considered to avoid further donor site morbidities, it bears the disadvantages of financial costs,^2^ concerns of tissue quality and biological incorporation,^3^ disease transmission,^4^ and possibly higher failure rates in the young high-risk active population. Among the autografts, BPTB (contralateral in this case) and hamstring tendons are popular choices, while quadriceps tendon has gained popularity in recent years for this purpose.^5^ Hamstring tendon harvest for ACLR may be associated with weakening the hamstring tendons anterior restraining action as an ACL protagonist in the already ACL-lesioned knee,^6^ and there is some evidence of higher revision rates compared to autologous BPTB^7^ and inferior functional outcome scores compared to quadriceps tendon.^8^ Thus, surgeons may prefer using contralateral BPTB or using quadriceps tendon in this setup of revision surgery, particularly in high-risk populations. Until today, the use of quadriceps tendon autograft has been described to our knowledge primarily as a soft tissue graft when revising failed BPTB ACLR,^5^^,^^9^ while quadriceps tendon–patellar bone has been described primarily for primary ACLR^10^ or for revision ACLR after failed ipsilateral hamstring autograft.^11^ In only 1 series, to our knowledge, using ipsilateral quadriceps tendon–bone for revising cases of failed BPTB ACLR was reported, but in that series, patients with persistent patella bone defect as a result of the previous harvest were precluded.^12^ This may be due to concerns of harvesting additional bone from the patella after bone harvest for the primary ACLR. In this article, we describe revision reconstruction of failed primary BPTB ACLR by using ipsilateral quadriceps tendon–bone autograft in the setup of persistent bone defect in the patella. This technique benefits several advantages: (1) using highly resilient graft tissue for revision ACLR in young active individuals; (2) bone-to-bone healing on the femoral side, which promotes fast biological incorporation; (3) additional length added to the tendinous part by harvesting patellar bone, which facilitates tibial tunnel fixation for surgeons who wish to avoid or are unfamiliar with the “all-inside” ACLR technique; and (4) a good alternative when the contralateral knee also undergoes BPTB ACLR or when the contralateral knee suffers significant patellar tendinopathy for surgeons who prefer using tendon-bone autograft in high-risk populations.

## Surgical Technique

Preoperative planning is based on the patient computed tomography (CT) as follows (Video 1):

1. The 3-dimensional CT images are observed, showing bilateral persistent patellar bone defects following bone–patellar tendon–bone primary ACLRs (Fig 1).

**Fig 1:**
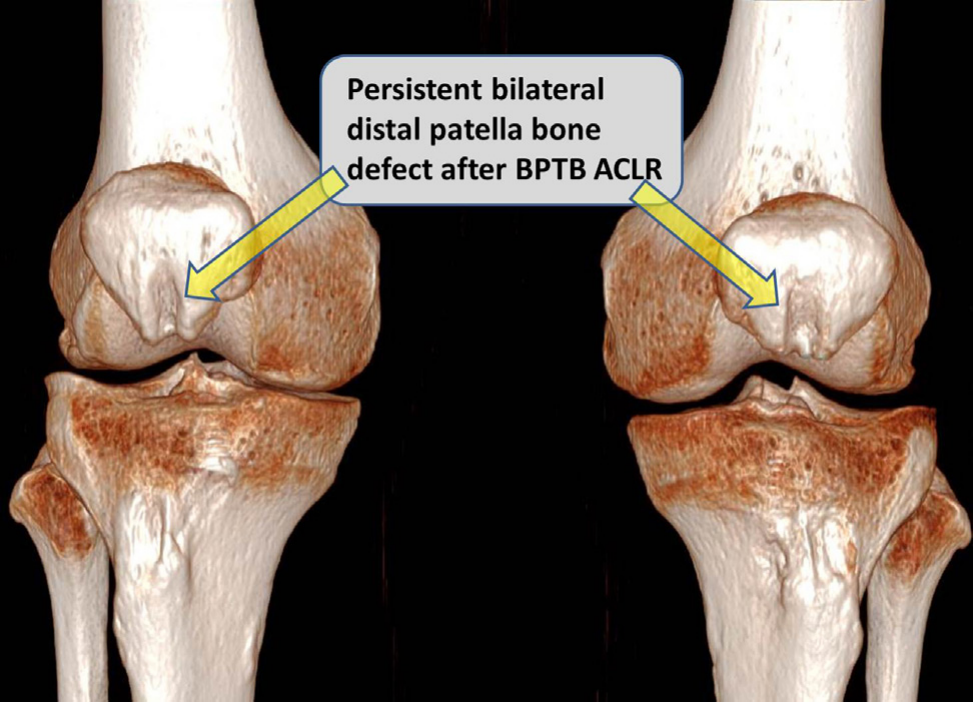
Three dimensional computed tomography images of right and left knees showing persistent patellar bone defects after bilateral autologous bone–patellar tendon–bone primary anterior cruciate ligament reconstructions.
2. Tunnel aperture locations are assessed. In this case, both seem appropriately located (Fig 2).

**Fig 2:**
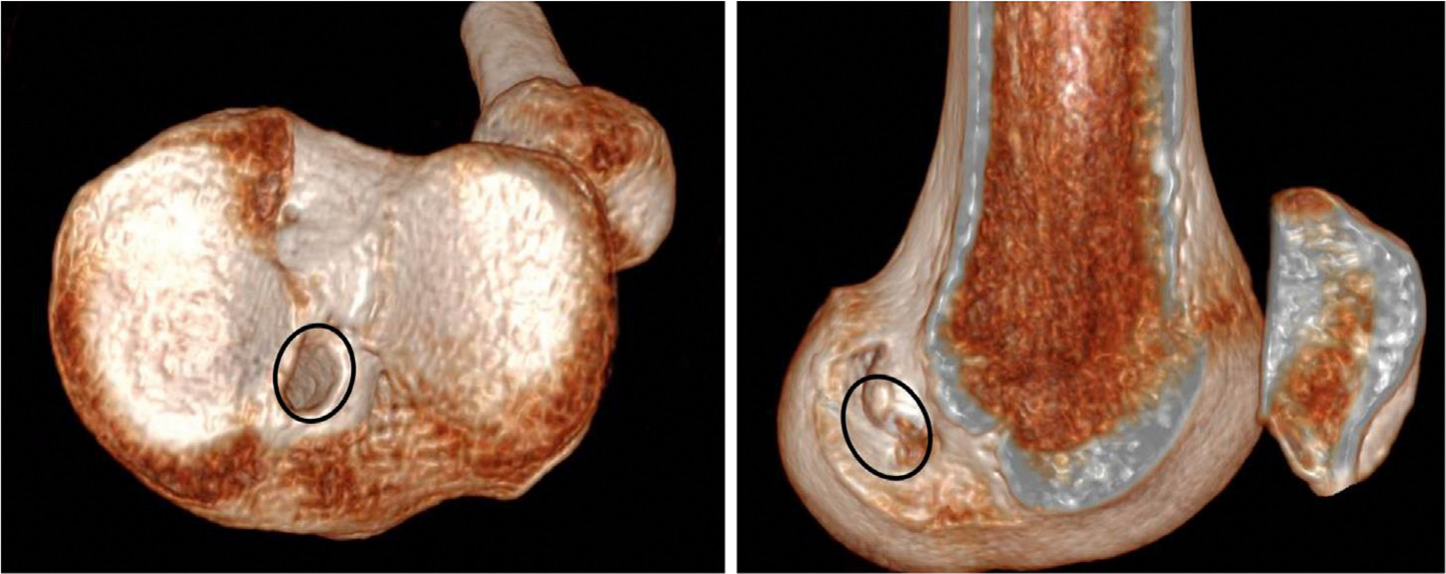
Three-dimensional computed tomography images of a left knee showing aperture locations of the tibia tunnel and femoral socket (black ovals). In this case, both seem appropriately located.
3. The axial CT cut confirms over 5 mm deep persistent distal patella bone defect (Fig 3).

**Fig 3:**
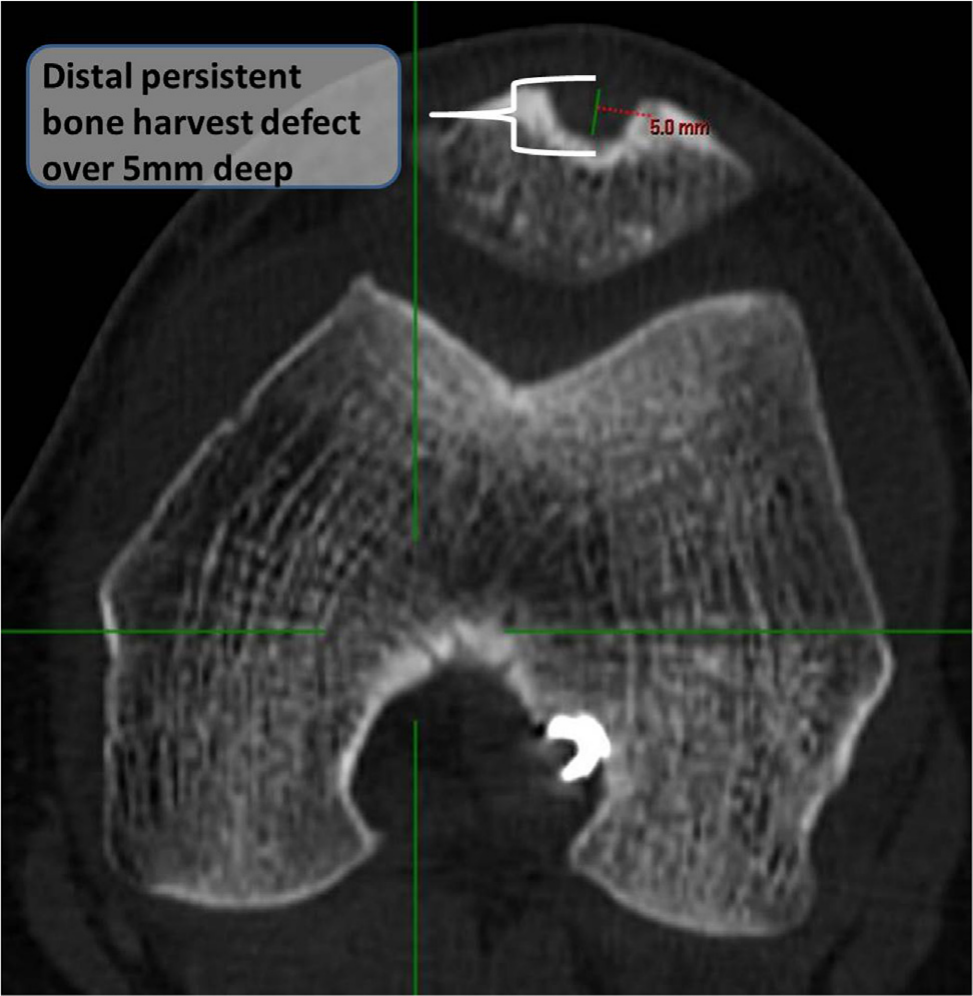
Two-dimensional axial computed tomography cut of a left knee showing over a 5-mm deep persistent distal patella bone defect, implying only partial fill of the harvest site after the primary surgery.
4. On the sagittal CT cut through the center of the distal bone defect, measurements are performed for planning harvesting a 15- to 18-mm-long proximal patellar bone plug and ensuring an 8- to 10-mm “safety” bone bridge remaining between the distal and proximal bone plug harvest sites (Fig 4).

**Fig 4:**
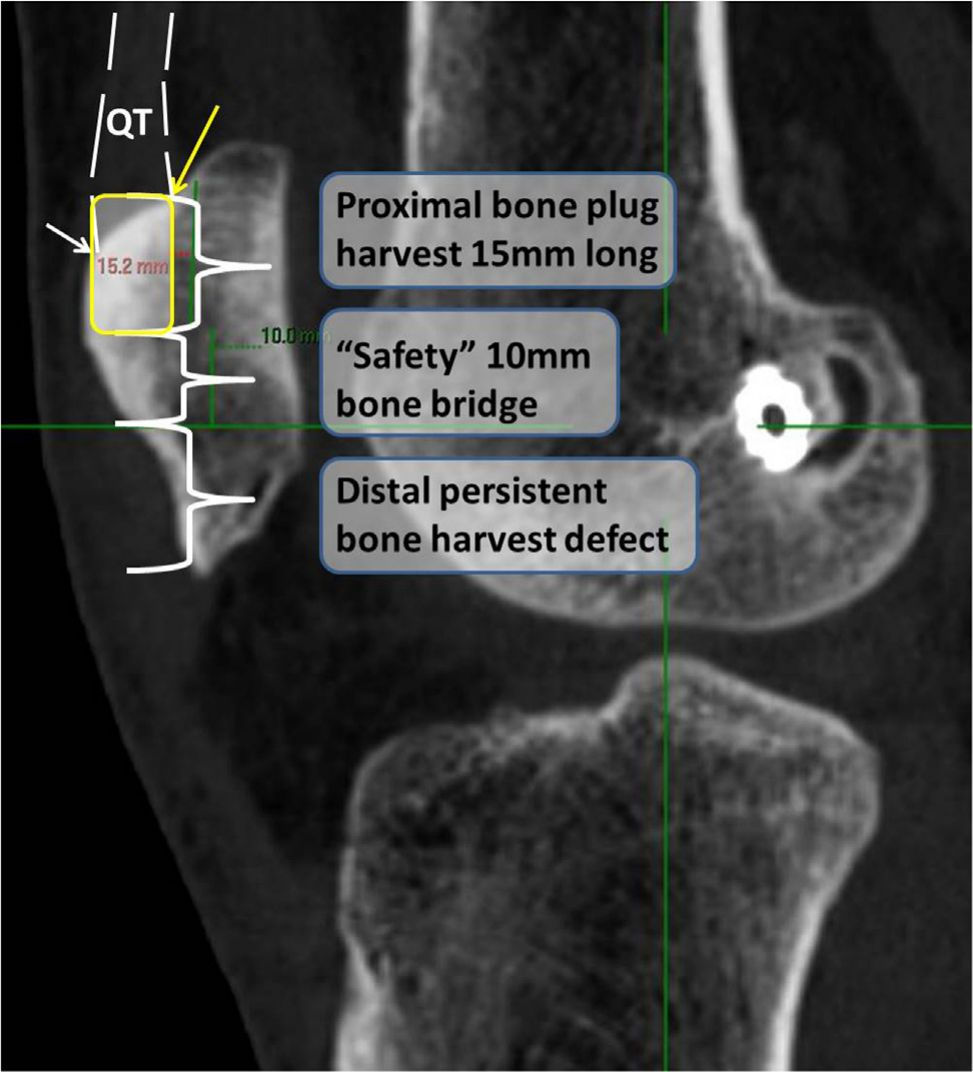
Two-dimensional sagittal computed tomography cut of a left knee through the center of the distal bone defect. Preoperative key measurements are performed for harvesting of a minimum 15-mm-long proximal patellar bone plug and ensuring an 8- to 10-mm “safety” bone bridge is remaining between the distal and proximal bone plug harvest sites. For this measurement, it is crucial to identify the deep insertion of the quadriceps tendon (yellow arrow) as the most proximal bony edge, which allows 5 mm or more of additional bone plug length compared to the superficial patella insertion of the quadriceps tendon (white arrow) and assists in avoiding cutting too distally when preparing the proximal patella bone plug.

In the operating room, prior to beginning surgery, the following steps are performed:

1. The patient is placed in a supine position. A tourniquet cuff (Zimmer) is placed around the proximal thigh but not inflated yet. A lateral thigh-post (Maquet) and a 3-L arthroscopic irrigation bag strapped distally onto the operating table and used as a foot bolster enable positioning and supporting the knee at 90° of flexion as well as mobilizing it freely from flexion toward extension during the operation as needed.
2. The knee is examined under anesthesia. Positive findings of the left knee in this case included grade 2+ Lachman and grade 2+ pivot-shift tests.

Surgical steps are then performed as follows:

1. All relevant skin markings are drawn, including quadriceps tendon harvest site, distal half of the previous patellar tendon harvest scar, Gerdy’s tubercle (GT), a line extending 7 cm proximally from GT for performing lateral extra-articular tenodesis (LET) with iliotibial band (ITB), and locations for anterolateral and anteromedial arthroscopic portals (Fig 5).

**Fig 5:**
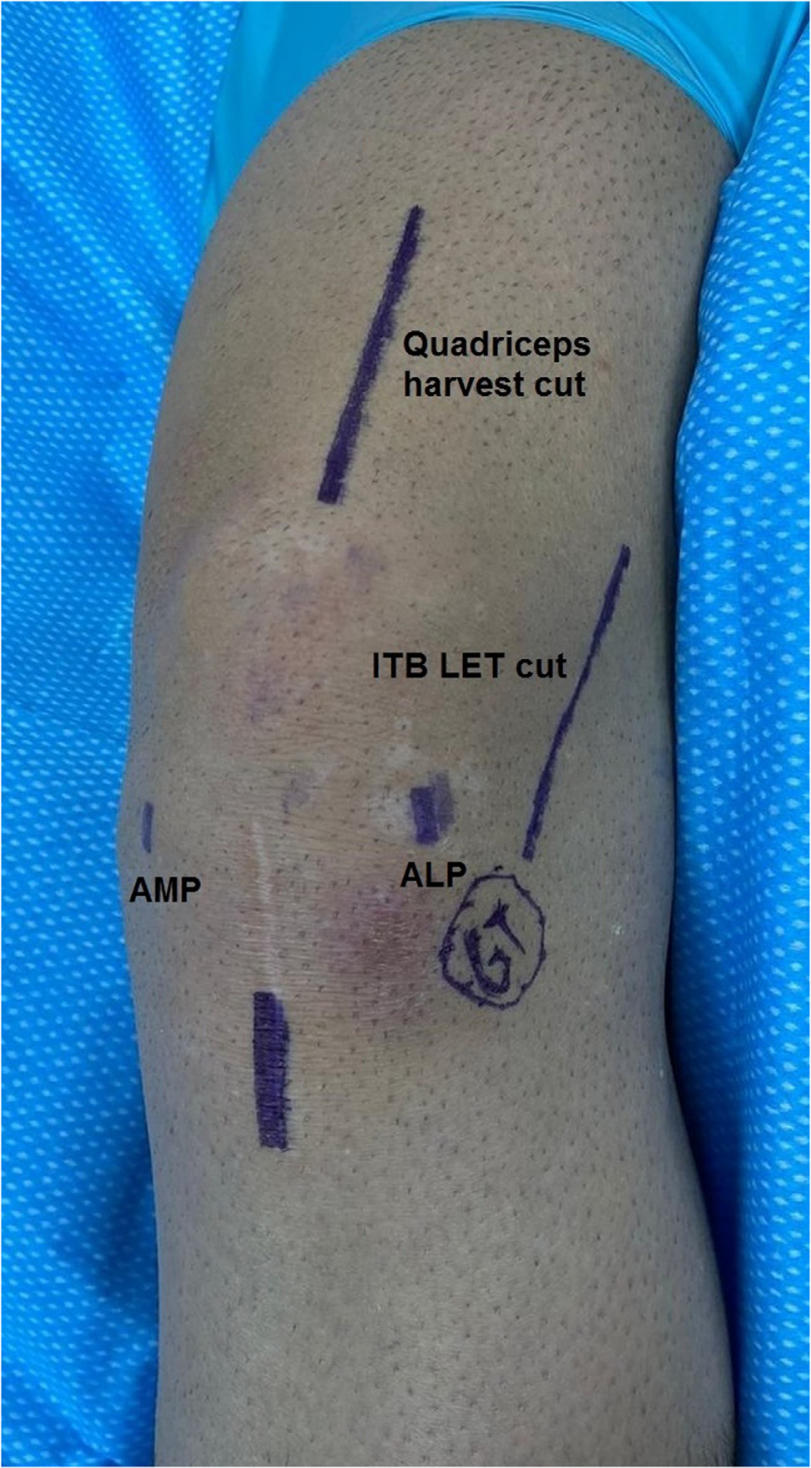
This is a left knee. All skin markings are drawn. (ALP, anterolateral portal; AMP, anteromedial portal; GT, Gerdy’s tubercle.)
2. Arthroscopic evaluation is performed with a 30° arthroscope (Olympus Inc) using standard anterolateral and anteromedial portals. In this case, a medial meniscus peripheral bucket handle fragment was displaced in the intercondylar notch with adhesions formed anteriorly (Fig 6) and anteromedially (Fig 7). These were gently resected using a 4.0-mm full-radius resector shaver (Stryker) (Fig 8). The periphery was gently rasped and the meniscus fragment reduced (Fig 9), confirming intact meniscus root and mechanical quality of the tissue for considering a later repair (Fig 10). At this point, the meniscus fragment was left alone to be repaired at the completion of the ACL graft passage later in order to avoid deep knee flexion after a massive meniscus repair. For surgeons who prefer repairing the meniscus before continuing to ACLR, the repair is completed at this step.

**Fig 6:**
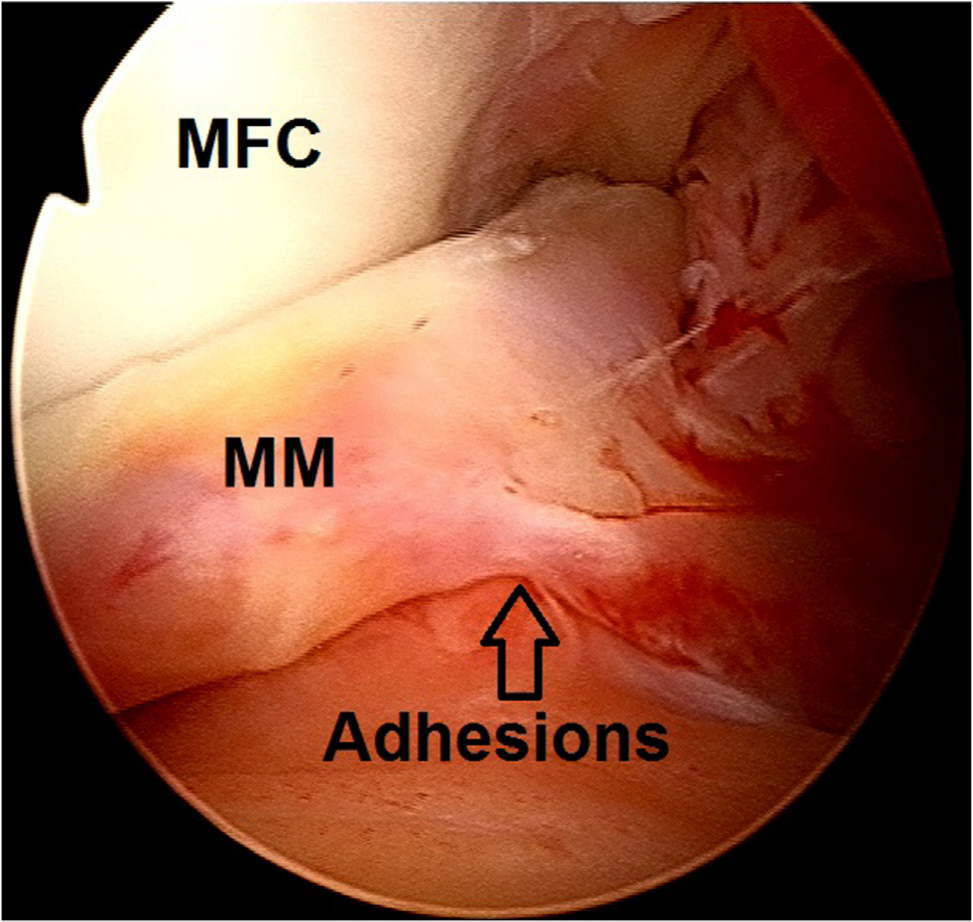
Arthroscopic view of a left knee using a 30° arthroscope (Olympus Inc) through the anterolateral portal. A chronically displaced medial meniscus bucket handle tear in the intercondylar notch with anterior adhesions is observed. (MFC, medial femoral condyle; MM, medial meniscus.)

**Fig 7:**
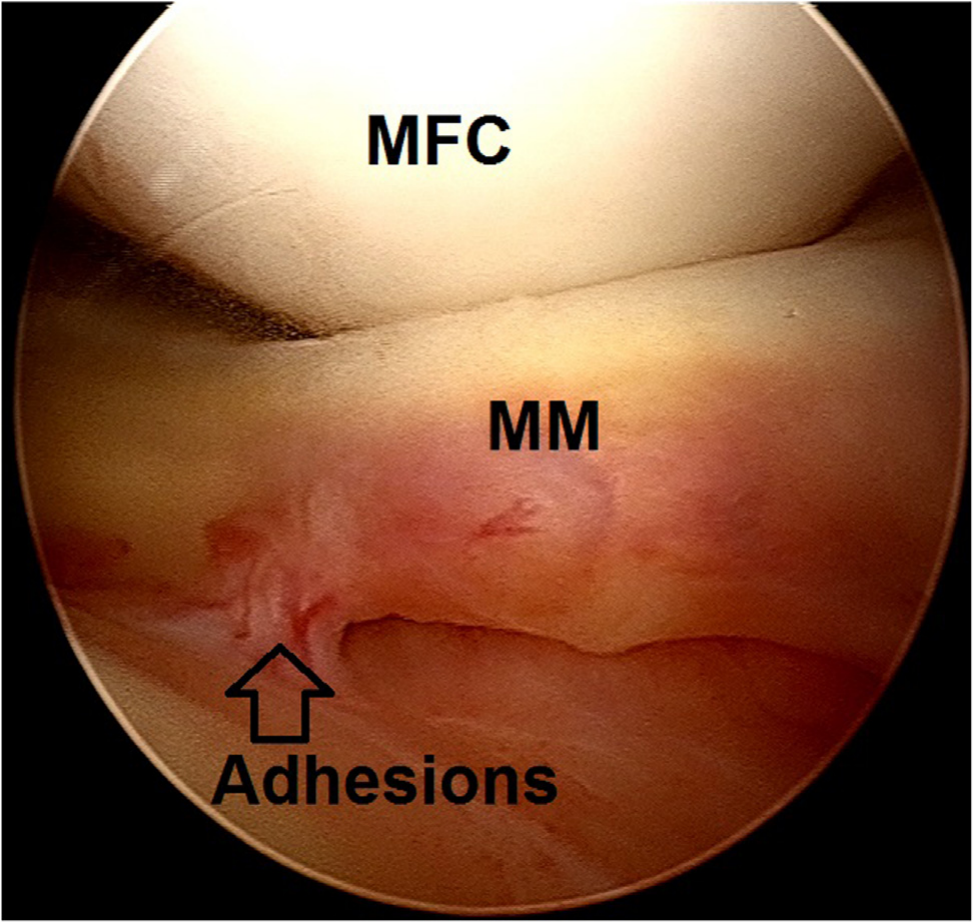
Arthroscopic view of a left knee using a 30° arthroscope (Olympus Inc) through the anterolateral portal. A chronically displaced medial meniscus bucket handle tear with anteromedial adhesions is observed.

**Fig 8:**
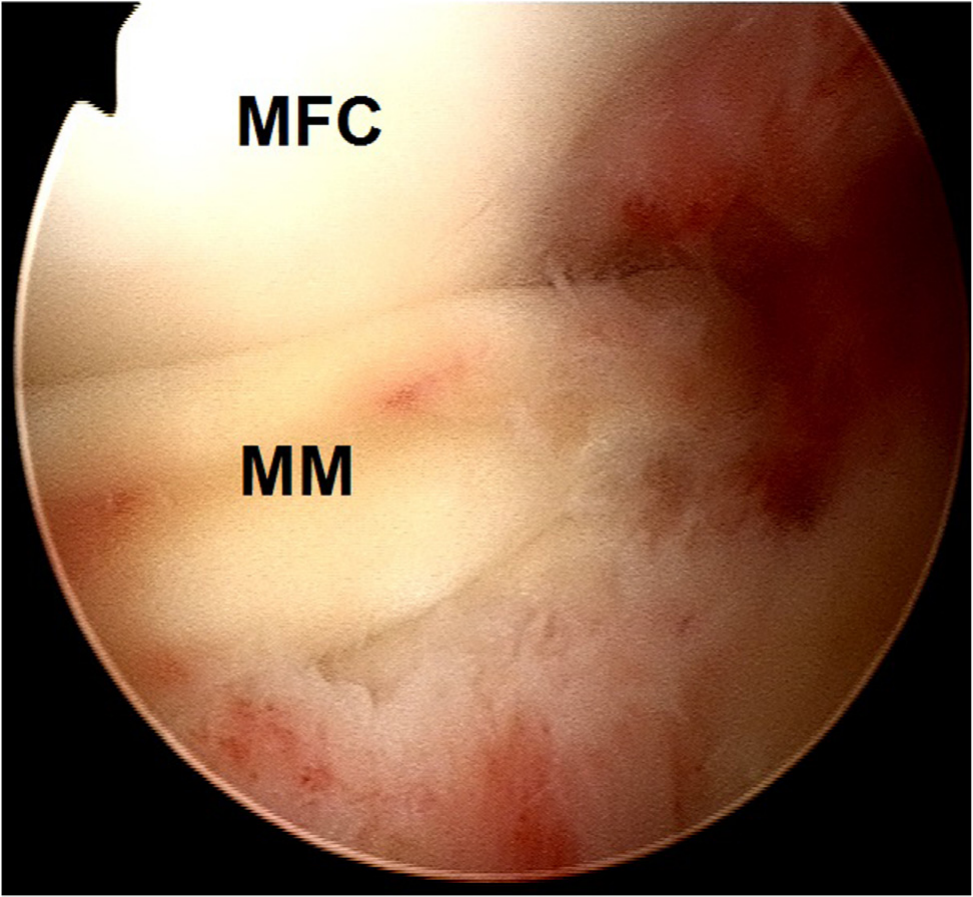
Arthroscopic view of a left knee using a 30° arthroscope (Olympus Inc) through the anterolateral portal. Anterior and anteromedial adhesions of a chronically displaced medial meniscus bucket handle fragment were gently resected using a 4.0-mm full-radius resector shaver (Stryker).

**Fig 9:**
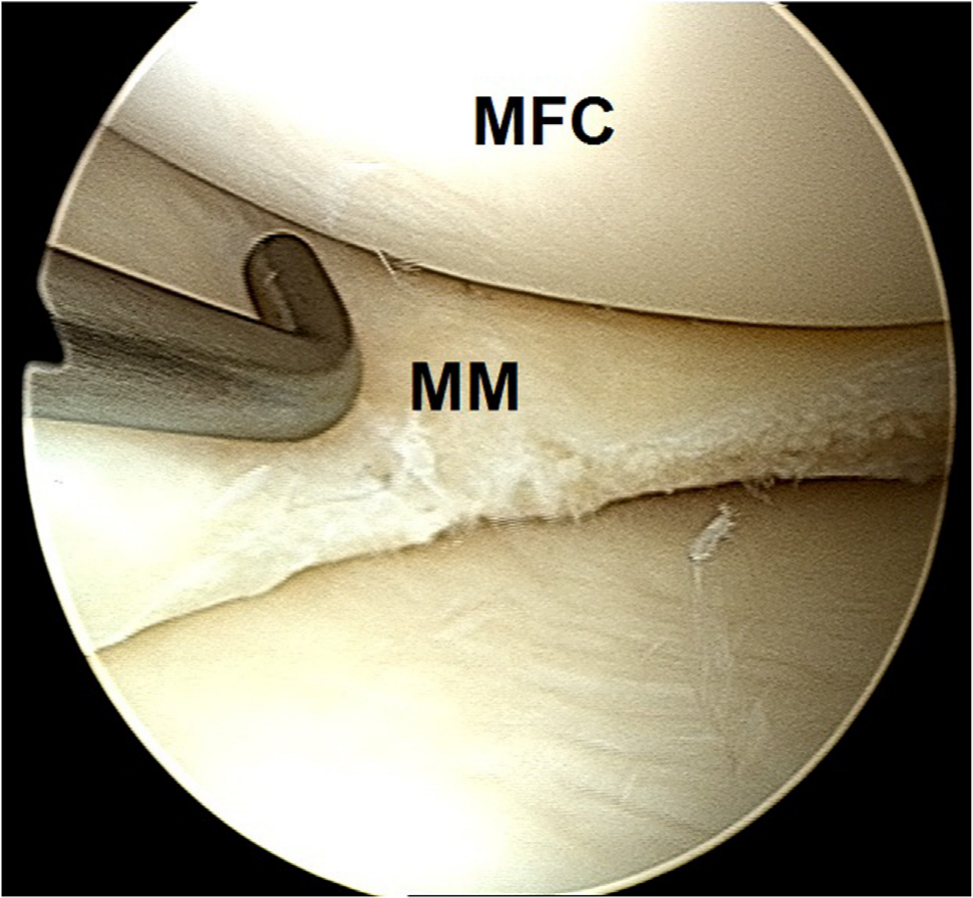
Arthroscopic view of a left knee using a 30° arthroscope (Olympus Inc) through the anterolateral portal. The periphery behind the meniscus fragment was gently rasped using a 4.0-mm full-radius resector shaver (Stryker) and the meniscus fragment reduced.

**Fig 10:**
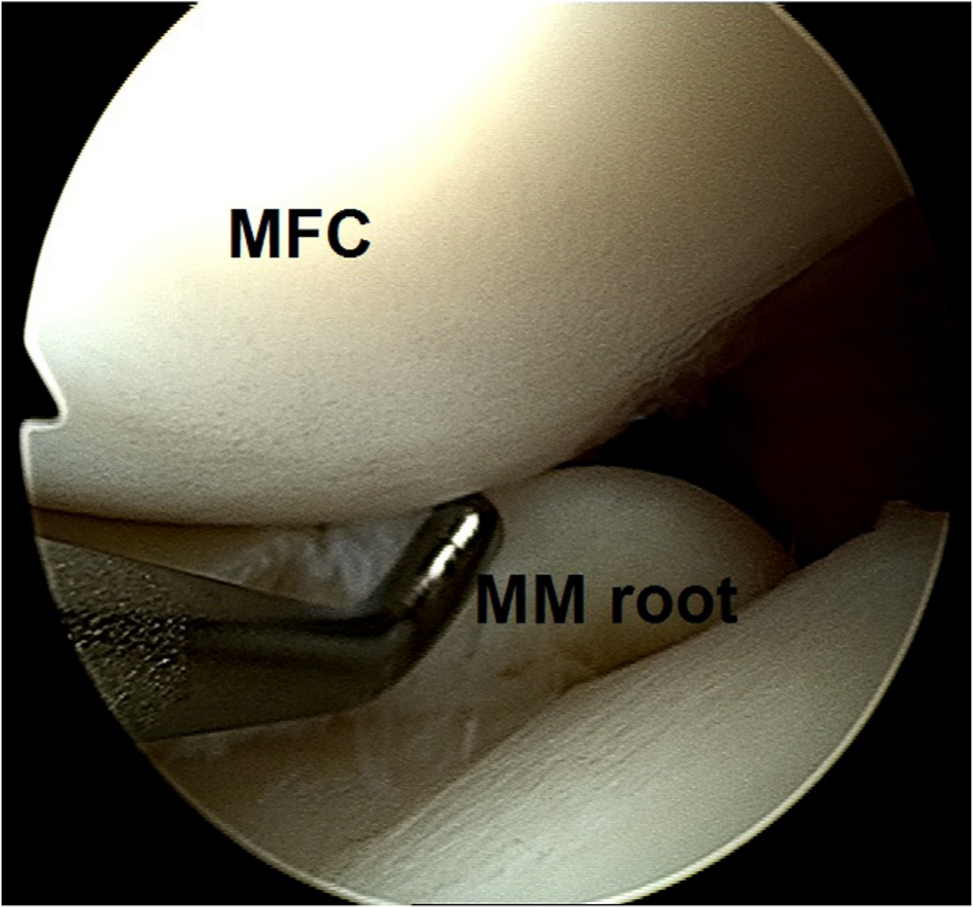
Arthroscopic view of a left knee using a 30° arthroscope (Olympus Inc) through the anterolateral portal, confirming intact meniscus root and mechanical quality of the tissue for considering a later repair.
3. A cyclops lesion in front of the failed BPTB graft is observed (Fig 11) and the insufficient BPTB graft tissue is probed (Fig 12).

**Fig 11:**
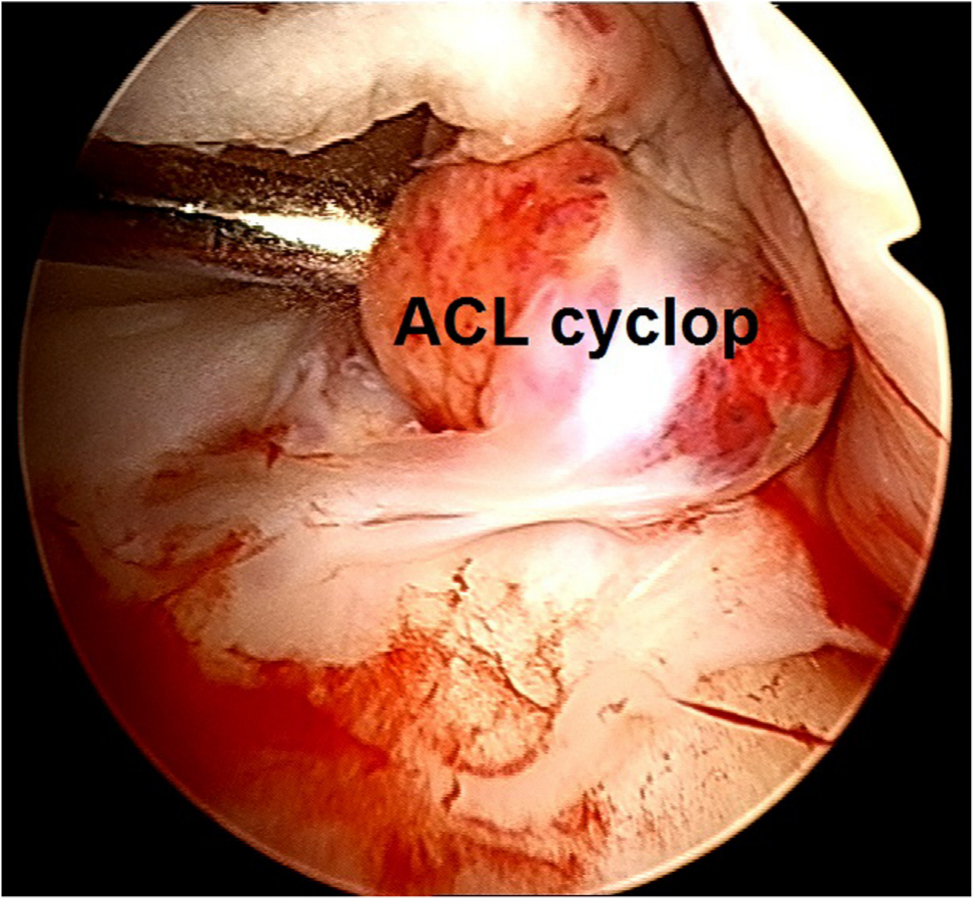
Arthroscopic view of a left knee using a 30° arthroscope (Olympus Inc) through the anterolateral portal. A cyclops lesion in front of the failed bone–patellar tendon–bone graft is observed.

**Fig 12:**
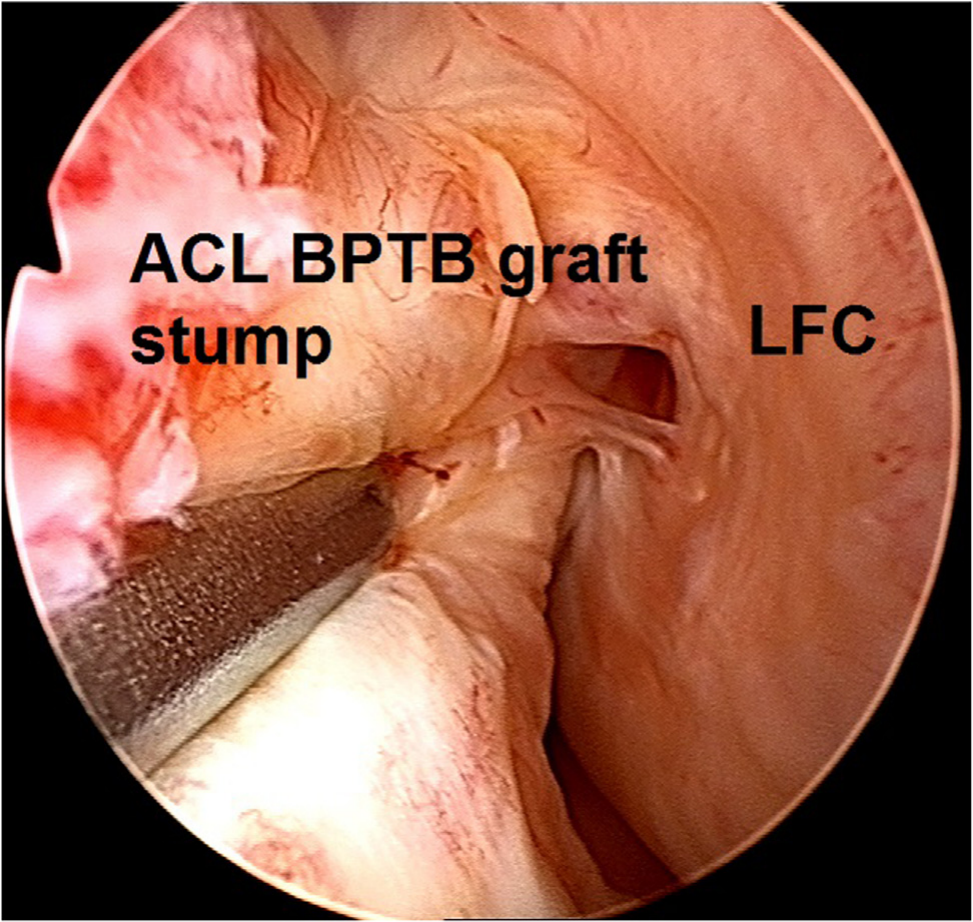
Arthroscopic view of a left knee using a 30° arthroscope (Olympus Inc) through the anterolateral portal. The insufficient bone–patellar tendon–bone graft tissue is probed.
4. The insufficient BPTB graft tissue is debrided using a 5.0-mm full-radius resector shaver (Stryker), and a gentle notchplasty is performed (Fig 13).

**Fig 13:**
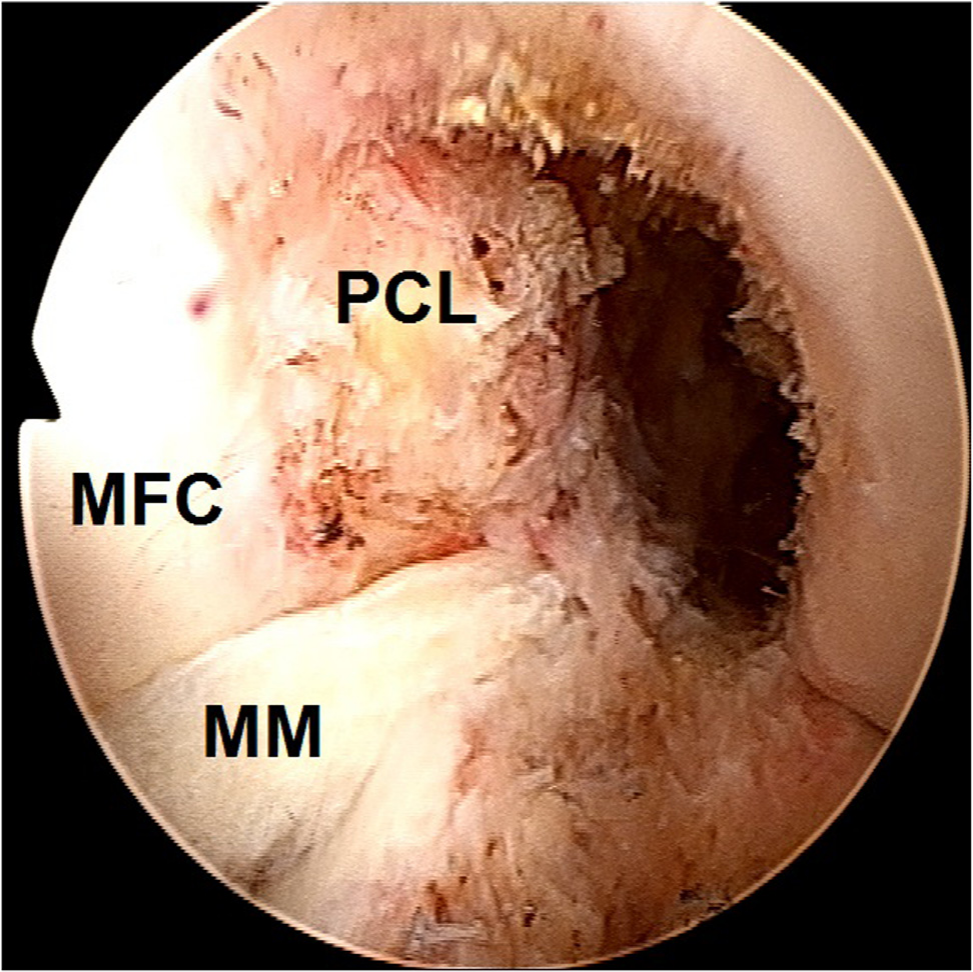
Arthroscopic view of a left knee using a 30° arthroscope (Olympus Inc) through the anterolateral portal. The insufficient bone–patellar tendon–bone graft tissue is debrided using a 5.0-mm full-radius resector shaver (Stryker) and a gentle notchplasty is performed.
5. A 7 × 20-mm titanium screw (Arthrex), used as the femoral fixation screw for the BPTB graft, is identified and removed using a screwdriver, preserving the screw socket (Fig 14).

**Fig 14:**
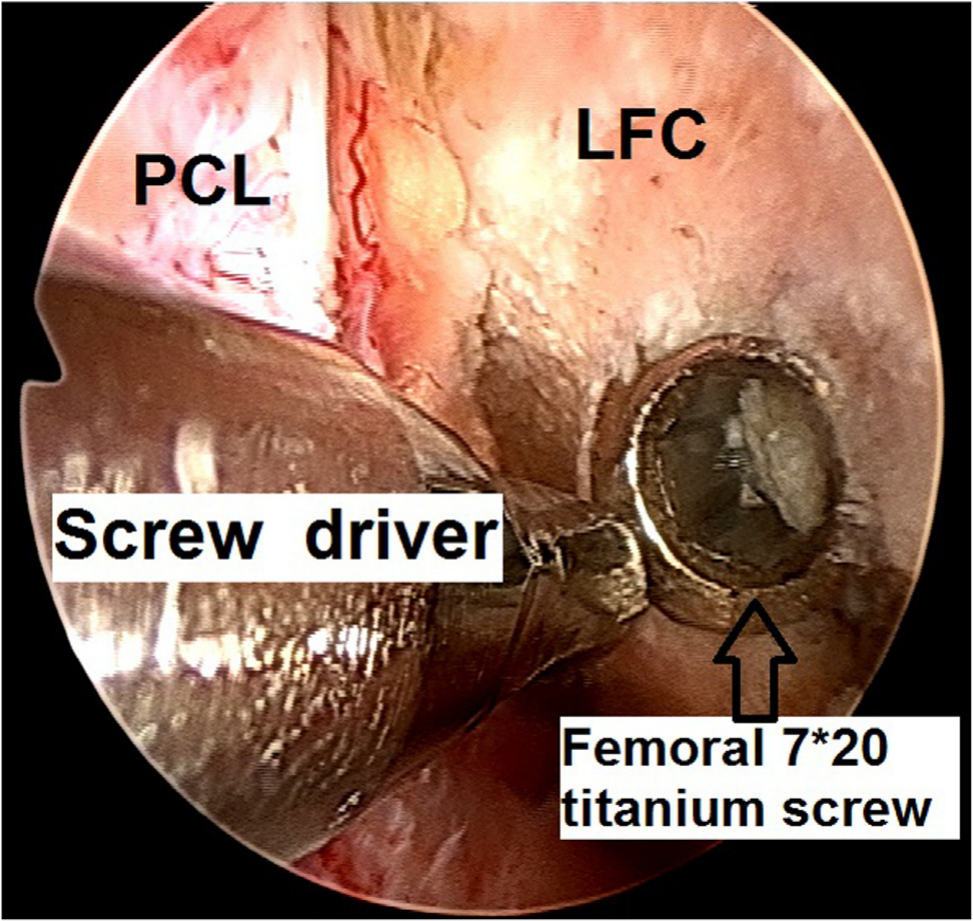
Arthroscopic view of a left knee using a 30° arthroscope (Olympus Inc) through the anterolateral portal. A 7 × 20-mm titanium screw (Arthrex), which was used as the femoral fixation screw for the bone–patellar tendon–bone graft, is identified and removed using a screwdriver, revealing the screw socket.
6. With the knee at 110° of flexion, a 7-mm over-the-top femoral offset guide (Arthrex) is inserted through an anteromedial portal, and a 2.4-mm guidewire (Arthrex) through this offset guide is inserted to an anatomic femoral ACL footprint location. Using a 9.5-mm diameter low-profile reamer (Arthrex) (Fig 15), an 18-mm-long femoral socket for the bone plug of the quadriceps graft is created, forming a “snowman configuration” socket, preserving the already existing femoral screw socket (Fig 16).

**Fig 15:**
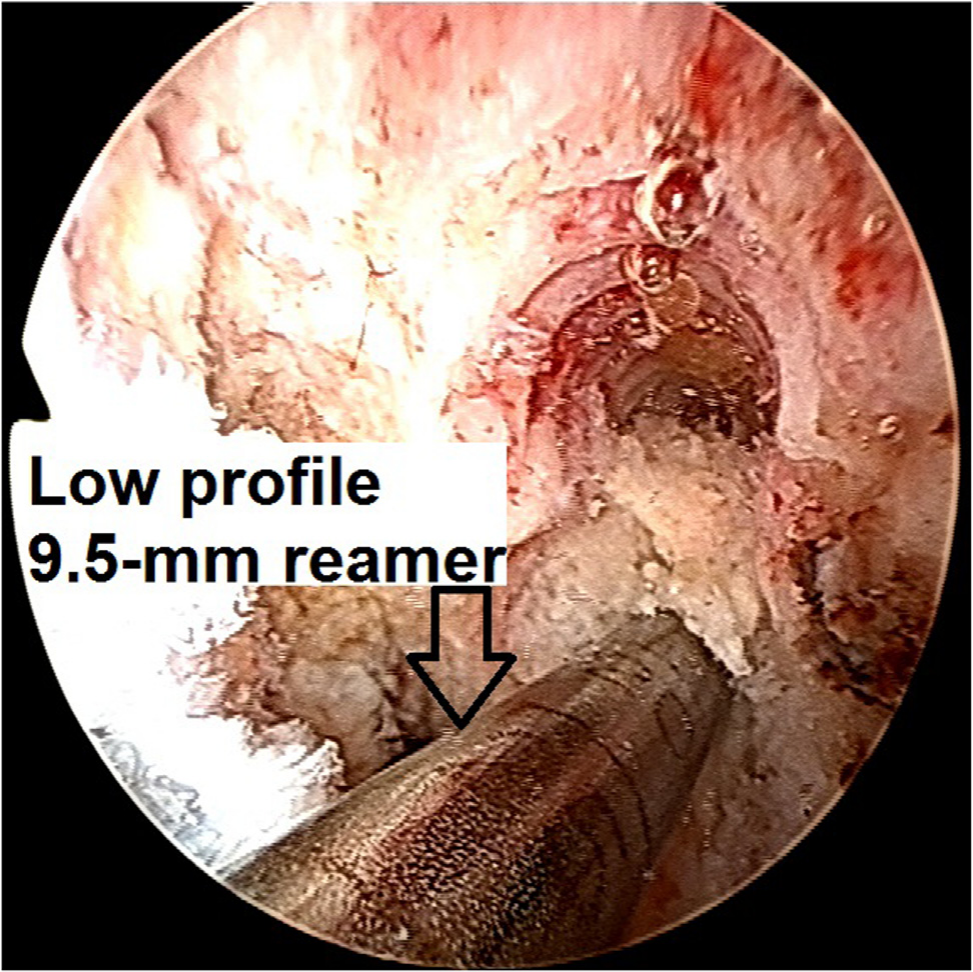
Arthroscopic view of a left knee using a 30° arthroscope (Olympus Inc) through the anterolateral portal. With the knee at 110° of flexion, a 7-mm over-the-top femoral offset guide (Arthrex) is inserted through an anteromedial portal, and a 2.4-mm guidewire (Arthrex) through this offset guide is inserted to an anatomic femoral ACL footprint location. Using a 9.5-mm diameter low-profile reamer (Arthrex), an 18-mm-length femoral socket for the bone plug of the quadriceps graft is created.

**Fig 16:**
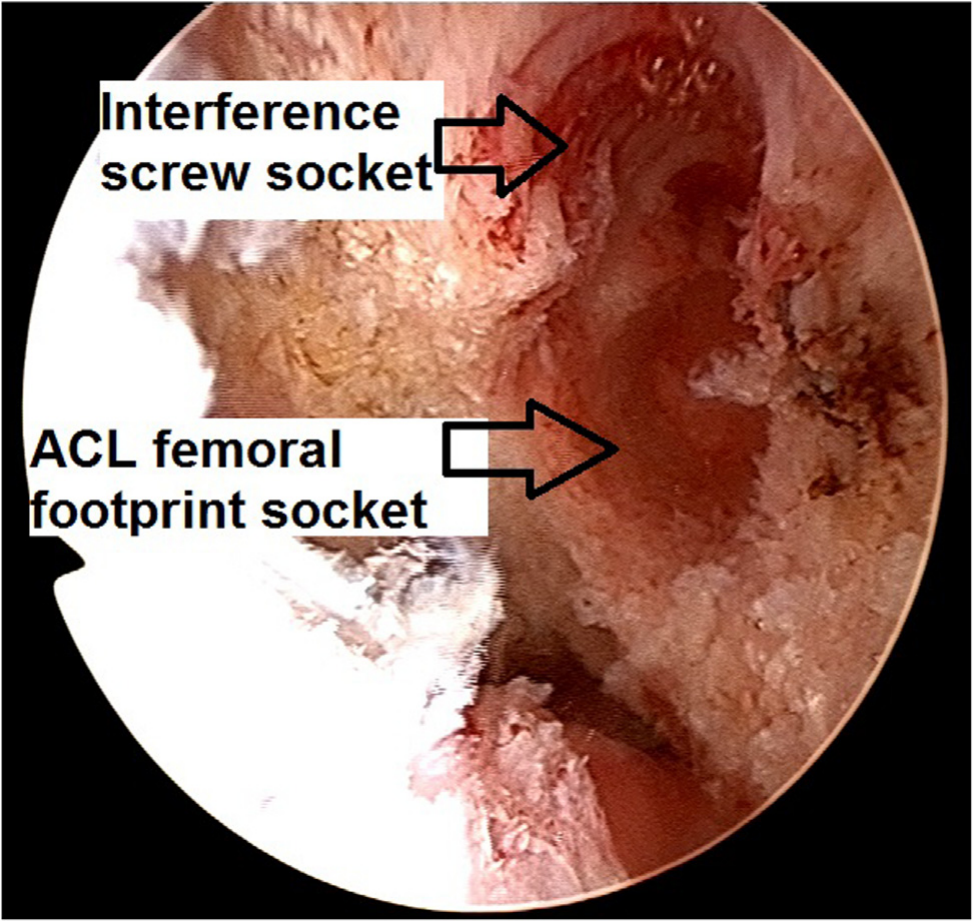
Arthroscopic view of a left knee using a 30° arthroscope (Olympus Inc) through the anterolateral portal. A “snowman configuration” socket in relation to the already existing femoral screw socket is created.
7. After removal of the existing 9 × 20-mm titanium interference screw (Arthrex) from the tibia, which was used for the primary BPTB ACLR, a 2.4-mm drill guide is inserted into the anatomic tibial ACL attachment area and is grasped with a clamp (Fig 17).

**Fig 17:**
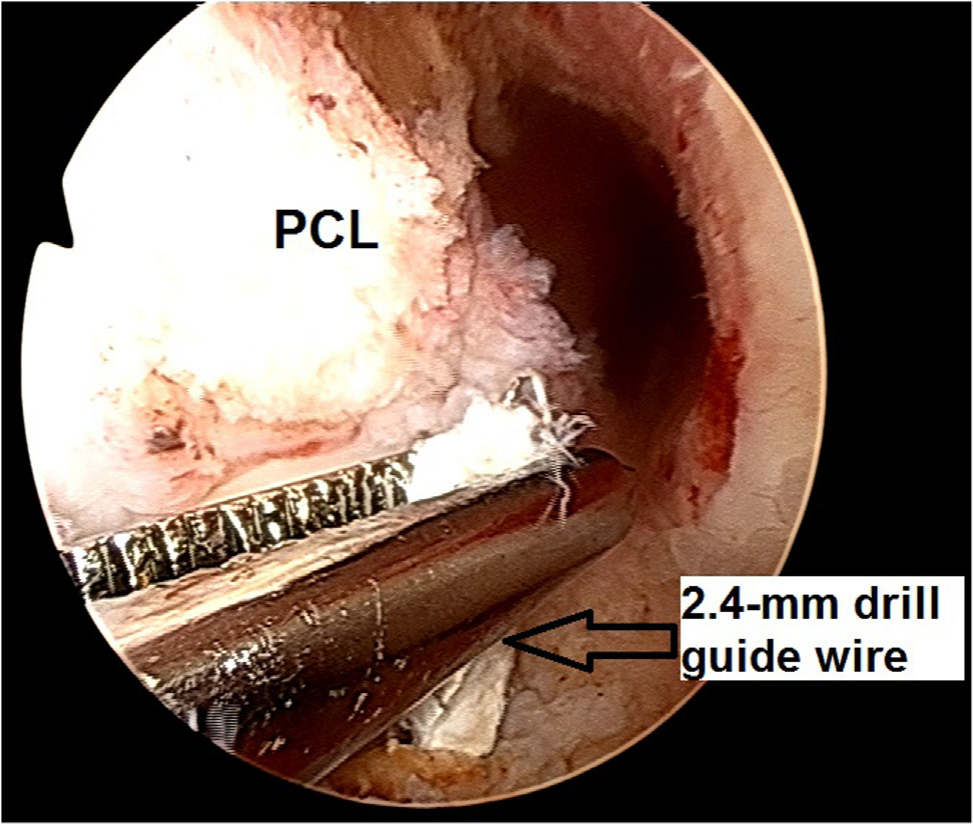
Arthroscopic view of a left knee using a 30° arthroscope (Olympus Inc) through the anterolateral portal. A tibial tunnel is created after the removal of the existing 9 × 20-mm titanium interference screw (Arthrex) from the tibia. A 2.4-mm drill guide is inserted into the anatomic tibial ACL footprint and is grasped with a clamp.
8. A 10-mm headed reamer (Arthrex) is inserted over the 2.4-mm drill guide and a 10-mm tibial tunnel is created, followed by cleaning the patellar tendon autograft remnants from the tunnel edges by using a 5.0-mm full-radius resector shaver (Stryker).
9. A shuttle suture is passed through the tibial tunnel and femoral socket (Fig 18), and then the medial meniscus bucket handle fragment is reduced to the anatomic location and repaired with multiple inside-out sutures as described previously (Fig 19).^13^

**Fig 18:**
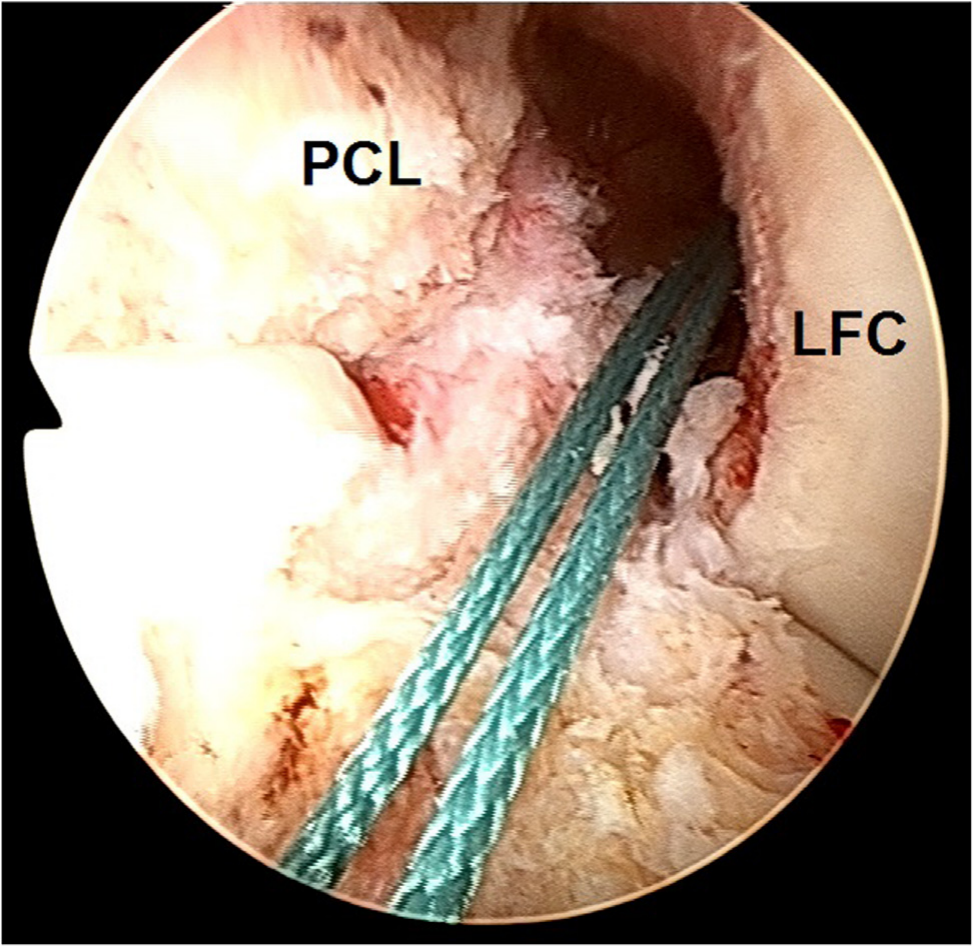
Arthroscopic view of a left knee using a 30° arthroscope (Olympus Inc) through the anterolateral portal. A shuttle suture is passed through the tibial tunnel and femoral socket. (LFC, lateral femoral condyle.)

**Fig 19:**
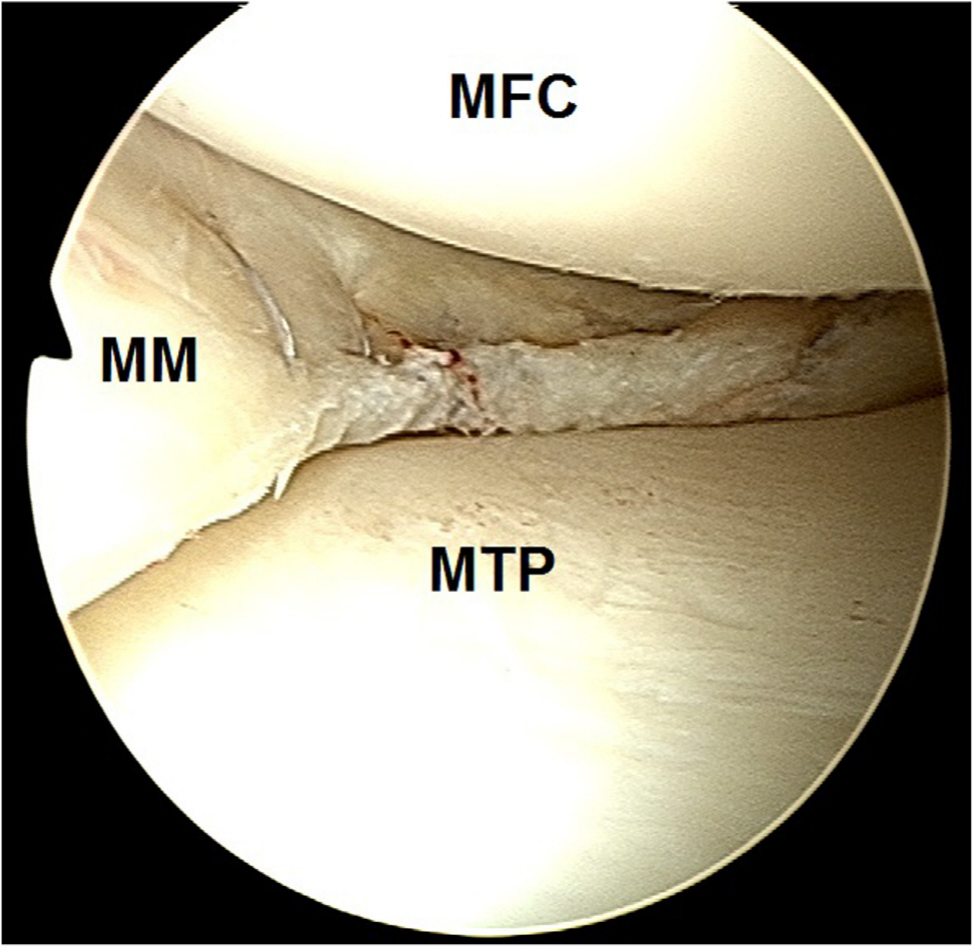
Arthroscopic view of a left knee using a 30° arthroscope (Olympus Inc) through the anterolateral portal. The medial meniscus bucket handle fragment is reduced to the anatomic location and repaired with multiple inside-out sutures as described previously.
10. Thigh tourniquet cuff (Zimmer) is now inflated to a pressure of 250 mm Hg.
11. Full-thickness ipsilateral quadriceps tendon harvesting begins, measuring 9 to 10 mm wide and 80 to 90 mm long. Harvest is initiated proximally and the tendinous part of the graft is whipstitched with a No. 5 Ethibond suture (Ethicon). A 15- to 18-mm-long proximal patella bone plug, in accordance with the preoperative CT planning, by 9 to 10 mm wide is measured and harvested, ensuring to leave an 8- to 10-mm-wide bone bridge between the persistent distal patellar bone deficiency site and the current proximal patellar bone plug harvest site (Fig 20). The tendinous part is lifted throughout bone plug harvest to identify and protect the deep attachment area of the quadriceps tendon to the patellar bone as a safety measure to avoid inadvertent violation of the deep tendon–bone insertion during bone plug harvest. The quadriceps harvest site is irrigated, and soft tissue layers are closed.

**Fig 20:**
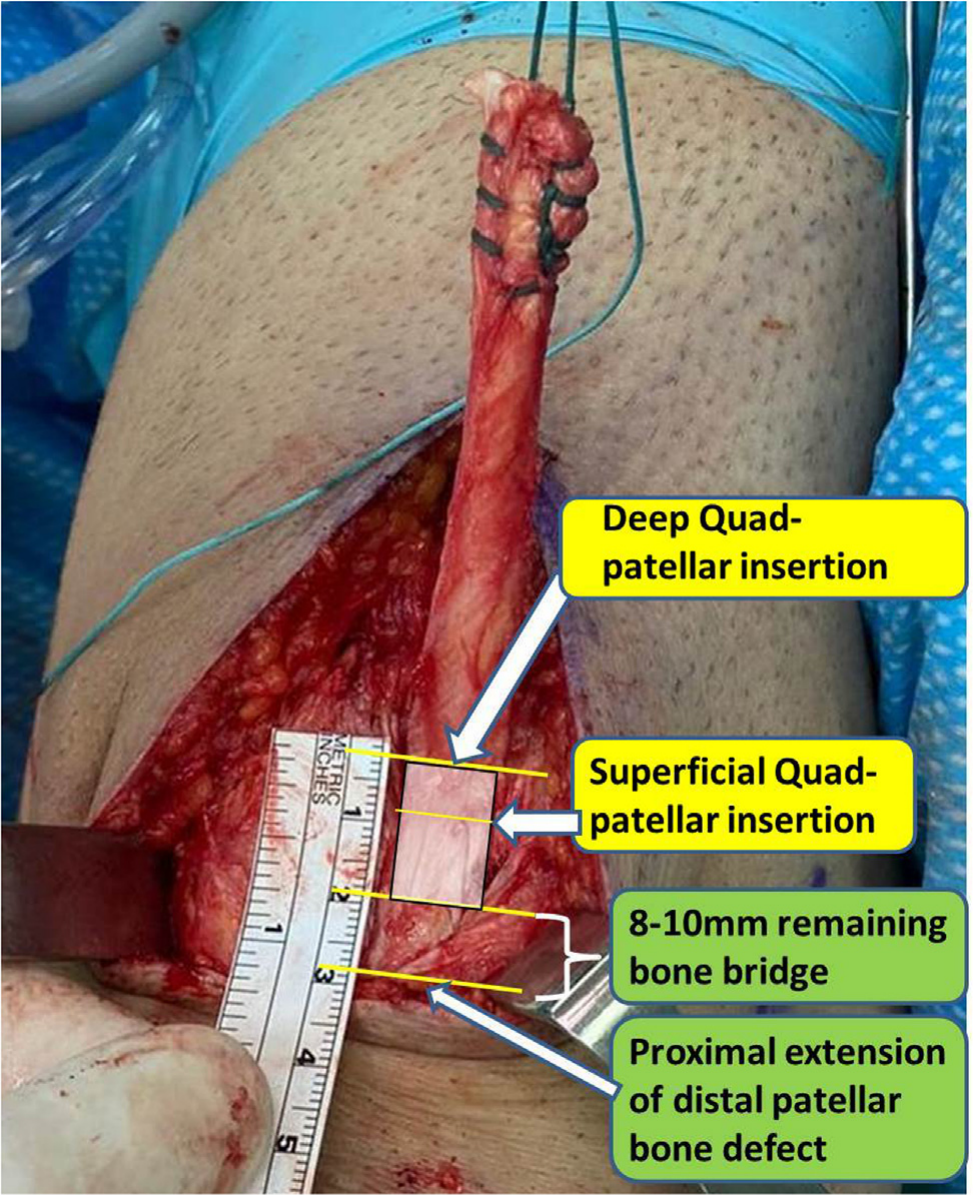
Ipsilateral quadriceps tendon harvest, measuring from 9 to 10 mm wide and 80 to 90 mm long. Harvest is initiated proximally and the tendinous part of the graft is whipstitched and lifted. All reference lines are identified for proper bone plug harvest according to the preoperative computed tomography measurements, including deep and superficial insertions of the quadriceps tendon to the proximal patellar pole, proximal extension of the distal patellar bone defect, and the planned distal extension of the quadriceps–patella bone plug, leaving a bone bridge between the 2 bone plug harvest sites.
12. The quadriceps tendon–bone autograft preparation is finalized on a side table. The bone plug is gently tapered with a Rongeur (Aesculap Inc) to smoothly fit the 9.5-mm femoral socket, and the tendinous part should smoothly fit the 10-mm diameter tibial tunnel. Two No. 5 Ethibond sutures (Ethicon) are passed in two 1.5-mm drill holes made in the patellar bone plug (Fig 21).

**Fig 21:**
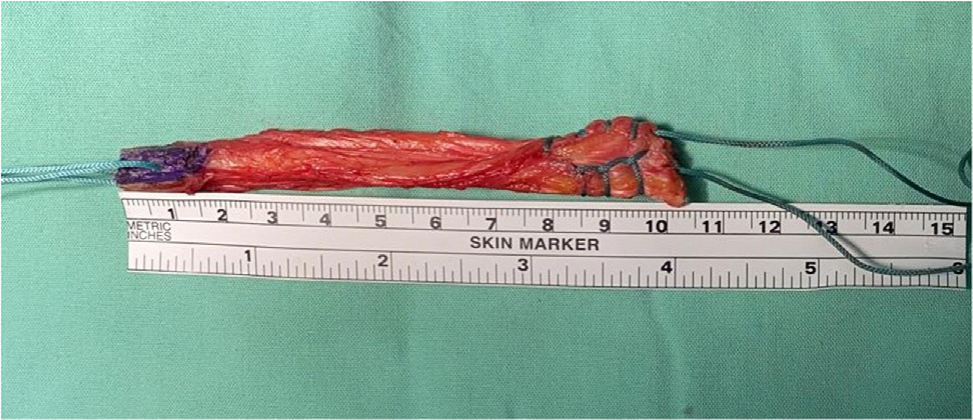
Finalized quadriceps tendon–bone autograft on a side table. The bone plug is colored in blue and measured 15 to 18 mm long. The tendon is whipstitched.
13. The quadriceps tendon–bone autograft is passed into the knee through the tibia tunnel and inserted into the femoral socket while aiming the bony margin of the bone plug toward the existing interference screw socket (Fig 22). This bone plug manipulation is important to avoid a later inadvertent placement of the metal interference screw against a tendinous part of the graft instead of against a bony margin, which could potentially harm the graft soft tissue.

**Fig 22:**
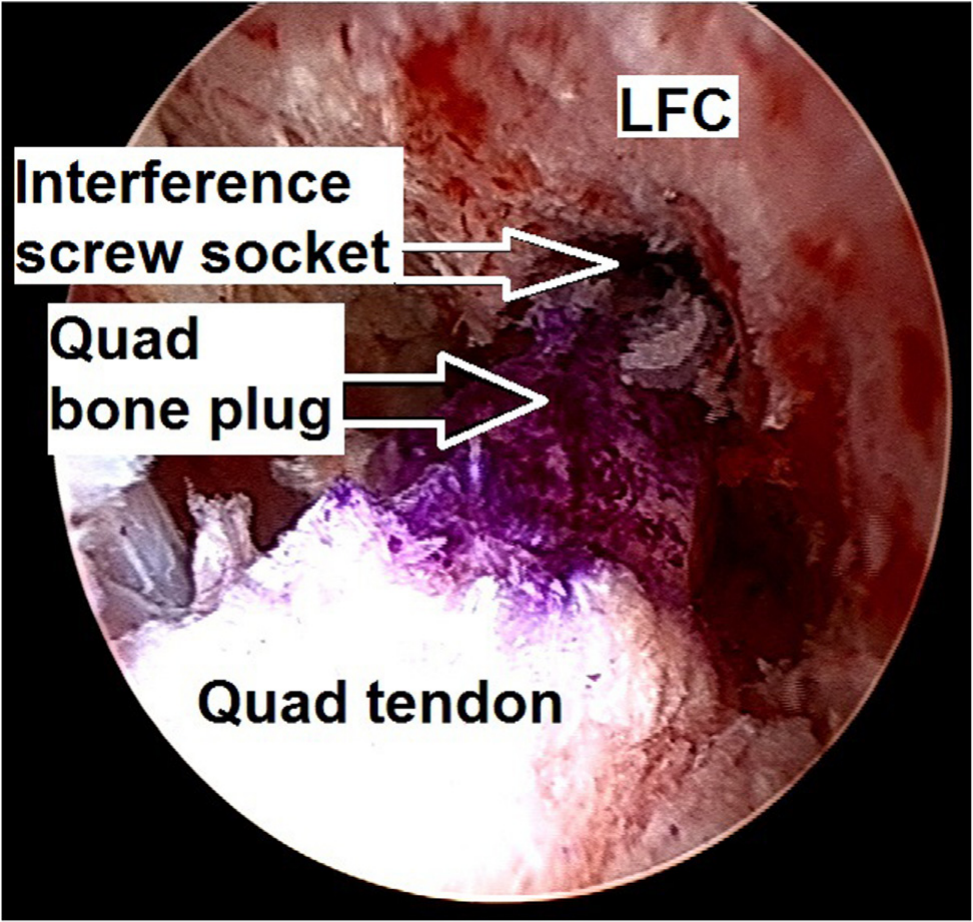
Arthroscopic view of a left knee using a 30° arthroscope (Olympus Inc) through the anterolateral portal. The quadriceps tendon–bone autograft is passed into the knee and inserted into the femoral socket while aiming the bony margin of the bone plug toward the planned interference screw socket.
14. The graft bone plug is fixed in the femoral socket with a 7 × 20-mm titanium interference screw (Arthrex), which is inserted into the already existing screw socket (Fig 23).

**Fig 23:**
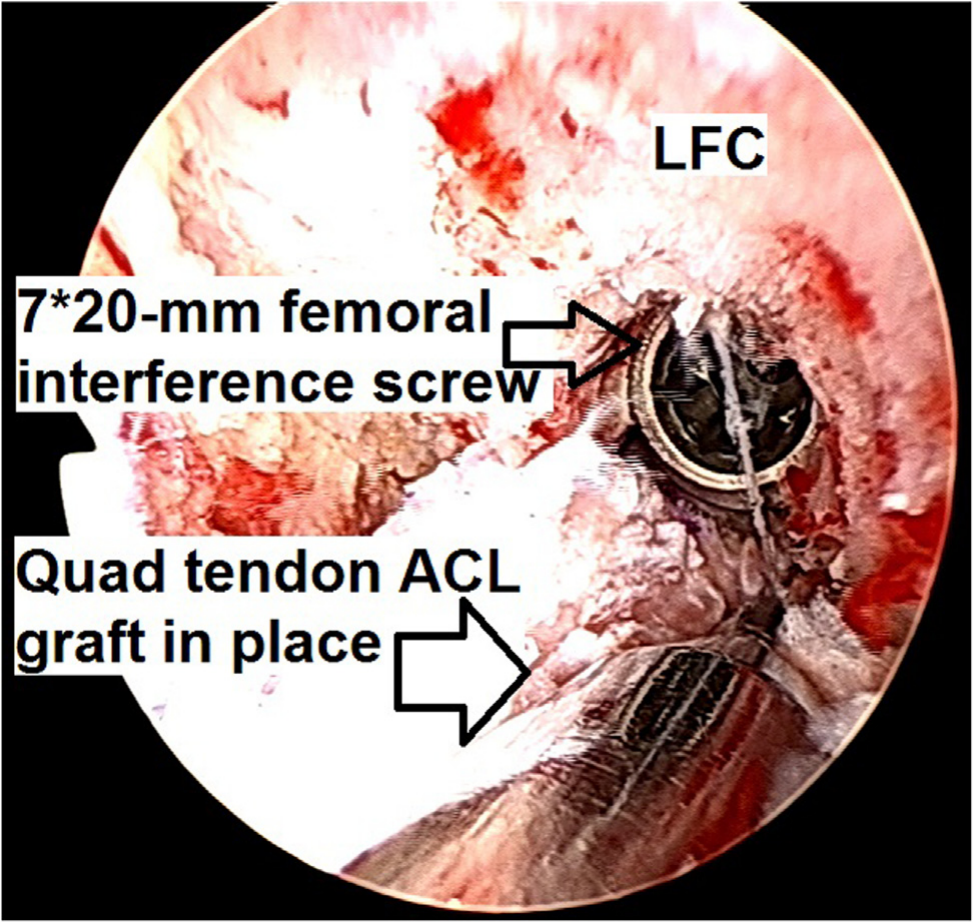
Arthroscopic view of a left knee using a 30° arthroscope (Olympus Inc) through the anterolateral portal. The graft bone plug is fixed in the femoral socket with a 7 × 20-mm titanium interference screw (Arthrex).
15. Proper graft location is finally confirmed (Fig 24) and the surgery continues on the extra-articular side.

**Fig 24:**
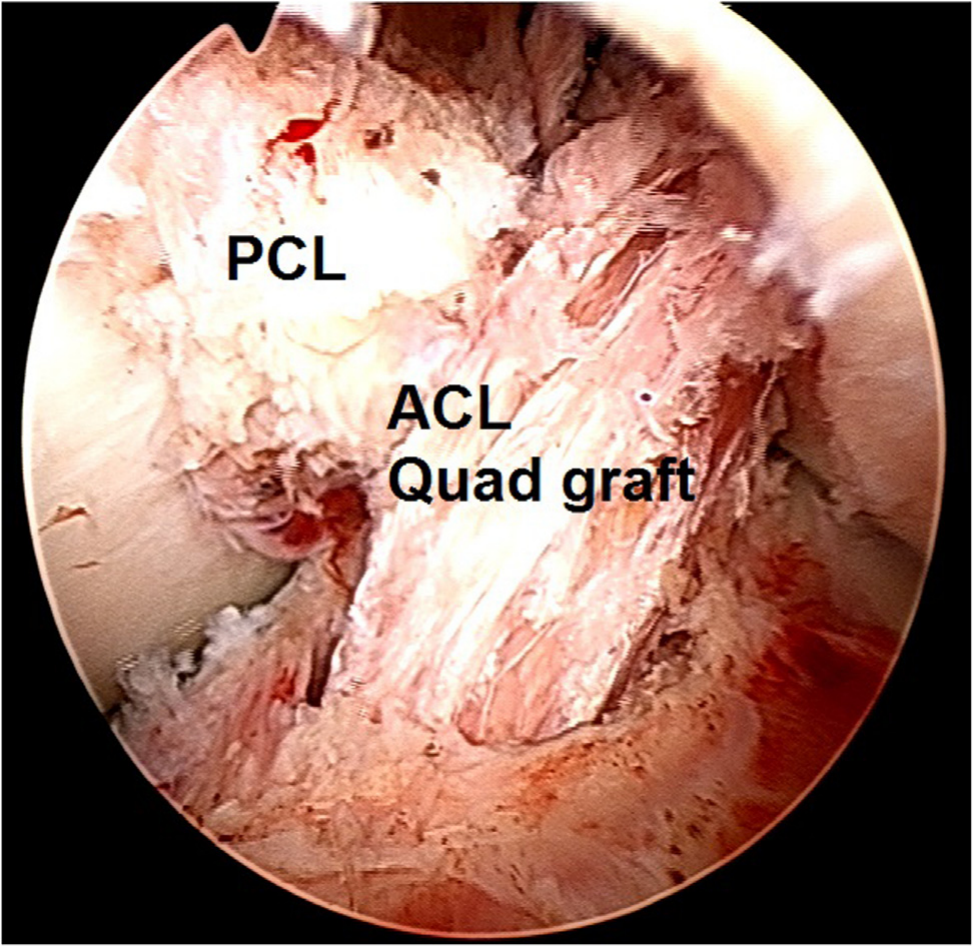
Arthroscopic view of a left knee using a 30° arthroscope (Olympus Inc) through the anterolateral portal. Final proper graft location is confirmed.
16. After cycling the knee with full tension applied to the graft, the tendinous part of the graft is fixed in the tibial tunnel with a 10 × 25-mm titanium interference screw (Arthrex) and backed up with a 8 × 20-mm titanium tendon staple (Arthrex) while the knee is held at full extension and maximum tension is applied to the graft.
17. A lateral extra-articular tenodesis is added using the ITB. The graft is fixed just posterior to the fibular collateral ligament (FCL) femoral insertion using a 3.5-mm double-loaded titanium suture anchor corkscrew (Arthrex) (Fig 25).

**Fig 25:**
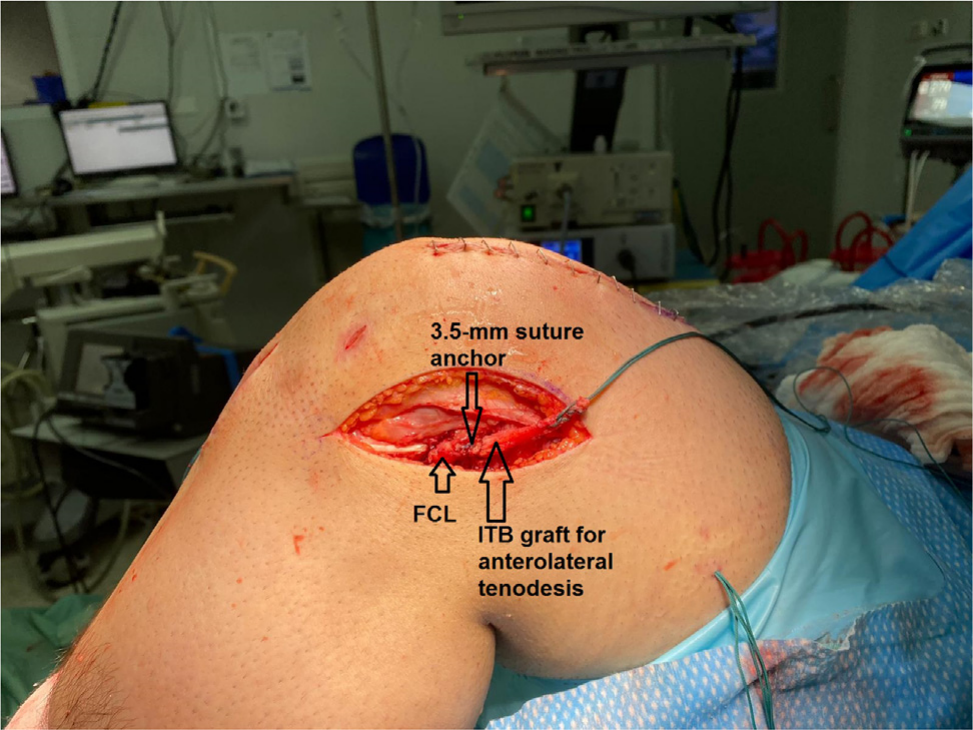
This is a left knee. A lateral extra-articular tenodesis is added using the iliotibial band (ITB). The graft is fixed just posterior to the fibular collateral ligament (FCL) femoral insertion using a 3.5-mm double-loaded titanium suture anchor corkscrew (Arthrex).

**Fig 26:**
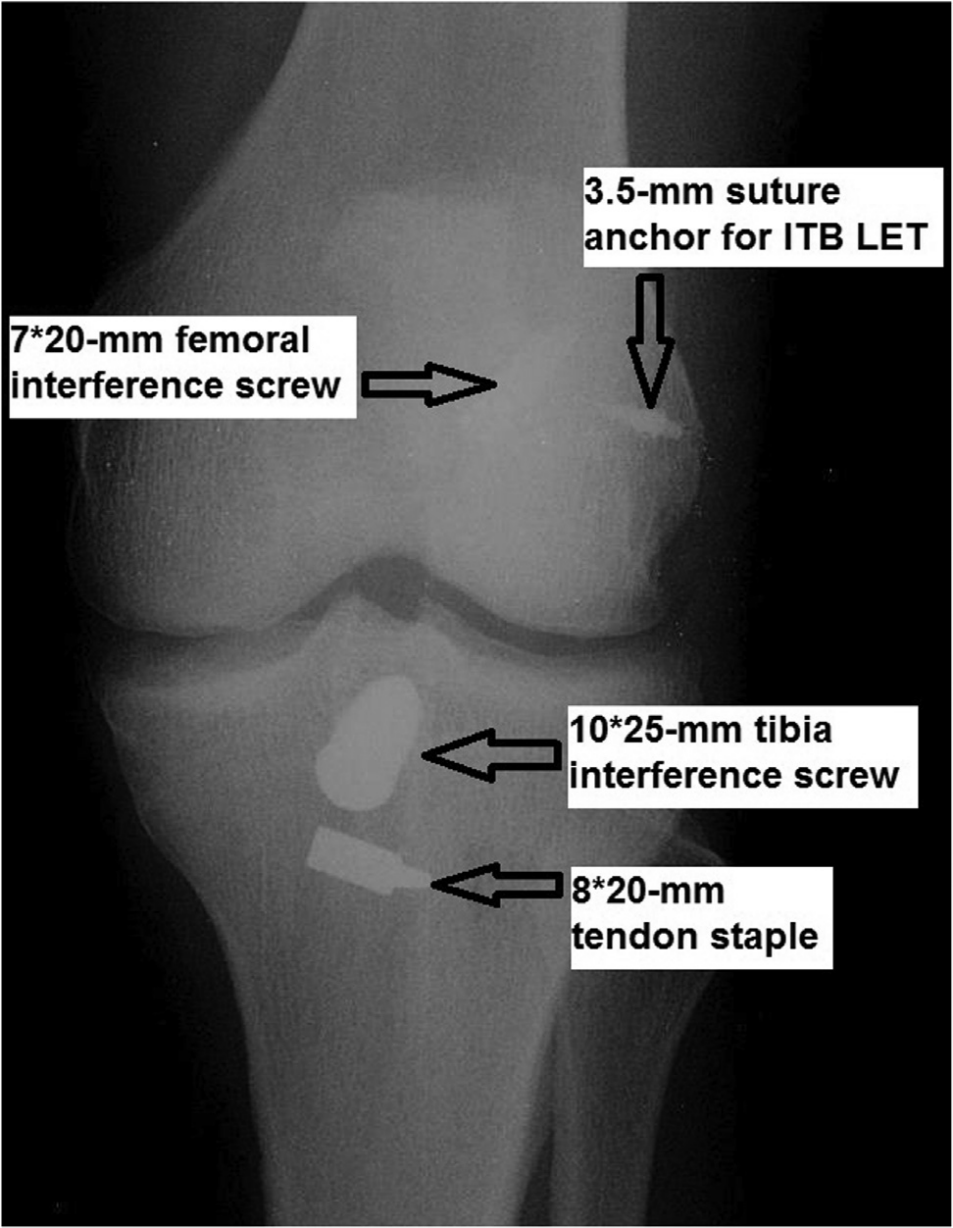
Postoperative anteroposterior radiograph of the left knee showing hardware locations and confirming patella integrity.

**Fig 27:**
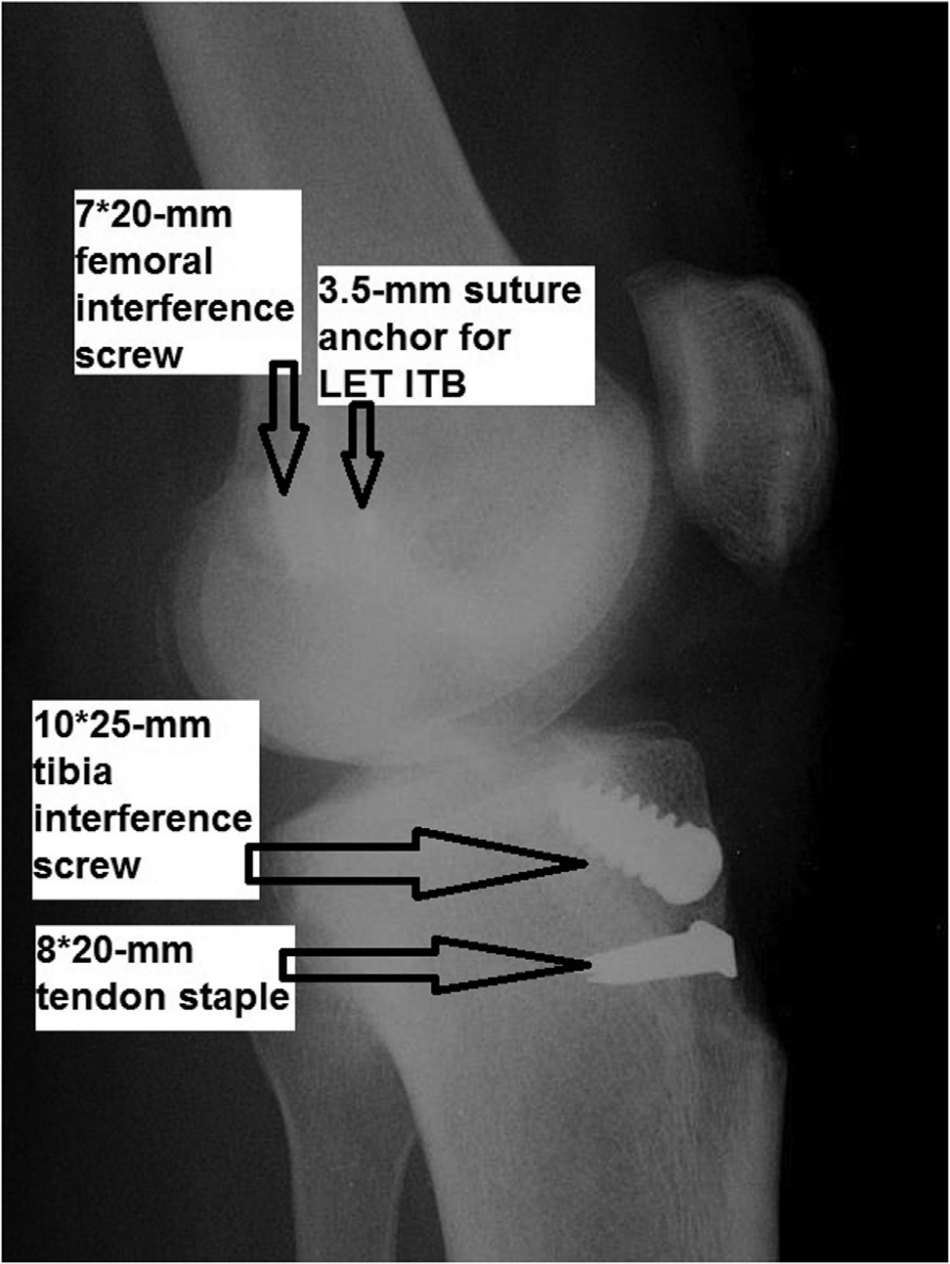
Postoperative lateral radiograph of the left knee showing hardware locations and confirming patella integrity.

Postoperative management:

1. Postoperative protocol includes wearing a T-scope knee brace (Breg Inc) without limiting range of knee motion and allowing full weightbearing. In case of a concomitant meniscus suture, range of motion is limited to 0° to 90° during the first postoperative month. Crutches are used until gait pattern is normalized. Closed kinematic chain exercises are encouraged during the rehabilitation with open chain avoided throughout the first 3 months after the operation to decrease potential anterior translation shearing forces.
2. At the first postoperative visit, 10 days after surgery, anteroposterior (AP) (Fig 26) and lateral (Fig 27) knee radiographs confirm patella integrity and hardware locations.

Table 1 summarizes tips and pearls for performing the operation.

**Table 1:** Tips and Pitfalls During Ipsilateral Quadriceps Tendon–Bone Revision ACL Reconstruction After Failed Primary BPTB ACL Reconstruction With Persistent Distal Patellar Bone Defect

Tips & Pitfalls During Surgery
**Tips**
• Accounting for the quadriceps–patellar bone deep insertion: During the CT-based preoperative planning as well as during surgery, it is important to account for the deep insertion of the quadriceps tendon into the patellar bone since this is where the proximal patellar bone plug practically starts. Failure to account for this anatomic detail, which adds about 5 mm length to the plug compared to the superficial insertion, may result in harvesting an unnecessary too long proximal bone plug, which may jeopardize patellar bone mechanical strength.
• Avoiding long tourniquet times: Thigh tourniquet cuff is not inflated during the arthroscopic procedure but only during the step of graft harvest.
• Femoral socket preparation: During femoral socket preparation, reusing the existing metal screw socket may be possible by creating a “snowman configuration.” In addition, reaming a 9.5-mm diameter new femoral socket for the graft bone plug, instead of a more common 10-mm socket, can decrease the risk of posterior socket wall breakage.
• Graft harvest: It is important to start the quadriceps harvest proximally instead of distally. This allows lifting the tendinous part first and identifying the quadriceps–patella bone deep insertion before marking the proximal patellar plug harvest lines.
• Graft passage and femoral fixation: Using the tip of a probe enables manipulating the bone plug into the femoral socket such that the free bony margin is facing the metal screw socket. This will avoid the metal screw later from inadvertently damaging the tendinous margin of the plug in the socket and weakening the construct. Alternatively, surgeons may use a PEEK 7 × 20-mm interference screw (Arthrex) if available, which is less abrasive to a tendinous tissue.
**Pitfalls**
• Harvesting a patellar bone plug longer than 15 to 18 mm may result in a narrow patella “safety” bone bridge between the proximal and distal patella plug harvest sites, while harvesting a bone plug shorter then 15 mm may result in a risky tendon graft abrasion in the femoral socket if using a titanium 7 × 20-mm screw.
• Femoral socket posterior wall breakage may not allow interference screw fixation. In this case, a cortical button can be a good fixation alternative.

ACL, anterior cruciate ligament; BPTB, bone–patellar tendon–bone; CT, computed tomography.

## Discussion

Based on the technique presented, surgeons who prefer using a compound of tendon-bone autograft for revision ACLR in the young athletic population should not be discouraged using ipsilateral quadriceps tendon–bone to address failed primary BPTB ACLR in knees with persistent distal patella bone harvest site defect. The principles presented, which include particular attention to the preoperative CT in addition to awareness of the specific quadriceps tendon–bone deep insertion location, which practically adds 5 mm length relative to the superficial insertion point, are keys in applying this technique safely and reproducibly. In addition, since an extensor mechanism autograft is used at the second time in this case, it is important to make sure that the patient has regained symmetric or nearly symmetric extensor torque before choosing to use again an extensor mechanism autograft. The major advantages, in addition to the high resiliency of the quadriceps tendon graft, include fast bone-to-bone biological incorporation on the femur, avoiding graft harvest morbidity in the unaffected contralateral limb, and cost-effectiveness of the procedure, which may be of importance in facilities where high-quality nonirradiated allograft tissue is not readily available. Disadvantages include the risk of weakening the patella bone with resultant fracture and further insult to the extensor mechanism, which may require long rehabilitation to regain extensor mechanism strength. Finally, for surgeons who may find this technique appealing but are concerned of adding proximal patella bone defect in cases of persistent distal patella defect, filling the proximal patella defect with autologous bone or bone substitute can be an option, although it may not be necessary.

## Supplementary Data

ICMJE author disclosure forms

Video 1 Preoperative computed tomography (CT) images show bilateral persistent distal patellar harvest site defects after autologous bone–patellar tendon–bone (BPTB) anterior cruciate ligament (ACL) reconstructions, appropriate femoral and tibial tunnel aperture locations on the left knee before ACL reconstruction revision surgery, and 8- to 10-mm-wide patella bone bridge remaining when planning harvesting 15- to 18-mm-long proximal patellar bone plug for ipsilateral quadriceps tendon–bone autograft, starting at the deep insertion site of the quadriceps tendon to the patella. Examination of the left knee under anesthesia shows grade 2+ Lachman and grade 2+ pivot-shift tests. Prior to starting the operation, all relevant skin markings are drawn. Arthroscopic evaluation using a 30° arthroscope is then performed. A medial meniscus peripheral bucket handle tear fragment is displaced in the intercondylar notch with adhesions formed anteriorly and anteromedially. These adhesions are gently resected, the periphery of the meniscus fragment is rasped, and the meniscus is reduced, confirming intact meniscus root and mechanical quality of the tissue for considering a later repair. At this point, the meniscus fragment is left alone to be repaired at the completion of ACL graft passage later in order to avoid deep knee flexion after a massive meniscus repair. A cyclops lesion and insufficient BPTB ACL graft tissue is debrided and a gentle notchplasty is performed. The ACL graft femoral fixation titanium screw is removed and the femoral socket is preserved. With the knee held at 110° of flexion, a 7-mm over-the-top femoral offset guide is inserted through an anteromedial portal, and a 2.4-mm guidewire is inserted to the anatomic femoral ACL footprint location. Using a 9.5-mm diameter low-profile reamer, an 18-mm-long femoral socket for the bone plug of the quadriceps graft is created, forming a “snowman configuration” socket that preserves the already existing femoral screw socket. After removal of the existing titanium interference screw from the tibia, which was inserted during the primary BPTB ACL reconstruction, a tibial tunnel is created with a 10-mm headed reamer. Tibial tunnel edges are cleaned and a shuttle suture is passed through the tibial tunnel and femoral socket. The medial meniscus bucket handle fragment is reduced to anatomic location and repaired with multiple inside-out sutures. Tourniquet cuff is inflated at this point, and a full-thickness ipsilateral quadriceps tendon graft harvesting begins, measuring 9 to 10 mm wide and 80 to 90 mm long. Harvest is initiated proximally and the tendinous part of the graft is whipstitched and lifted. A 15- to 18-mm-long proximal patella bone plug, in accordance with the preoperative CT measurement, by 9 to 10 mm wide is measured and harvested, ensuring to leave an 8- to 10-mm-wide bone bridge between the persistent distal patellar bone plug deficiency site and the current proximal patellar bone plug harvest site. The tendinous part is lifted throughout bone plug harvest to identify and protect the patellar bone–quadriceps deep attachment site as a safety measure to avoid inadvertent violation of the deep tendon–bone insertion during bone plug harvest. The quadriceps harvest site is irrigated and soft tissue layers are closed. The quadriceps tendon–bone autograft is finalized on a side table. The quadriceps tendon–bone autograft is passed into the knee and inserted into the femoral socket, aiming the bony margin of the bone plug toward the existing interference screw socket. The graft bone plug is fixed in the femoral socket with a 7 × 20-mm titanium interference screw (Arthrex). Proper ACL graft location is confirmed and the operation continues on the extra-articular side. After cycling the knee with full tension applied to the graft, the tendinous part of the graft is fixed in the tibial tunnel with a 10 × 25-mm titanium interference screw (Arthrex) and backed up with an 8 × 20-mm titanium tendon staple (Arthrex) while the knee is held at full extension and maximum tension is applied to the graft. A lateral extra-articular tenodesis is then added using an ITB strip. The ITB graft is fixed just posterior to the FCL femoral insertion using a 3.5-mm double-loaded titanium suture anchor corkscrew (Arthrex) with the foot held at neutral rotation. All layers are closed and the revision reconstruction is complete. Postoperative radiographs confirm hardware locations and patella integrity.
